# Effect of Probiotic Dose Escalation on Gut Microbiota and Clinical Outcomes in Preterm Infants—A Systematic Review

**DOI:** 10.3390/children10101710

**Published:** 2023-10-20

**Authors:** Chandra Rath, Gayatri Athalye-Jape, Shripada Rao, Sanjay Patole

**Affiliations:** 1 Neonatal Directorate, King Edward Memorial Hospital for Women, Subiaco, WA 6008, Australia; chandra.rath@health.wa.gov.au (C.R.); gayatri.jape@health.wa.gov.au (G.A.-J.); 2 Neonatal Directorate, Perth Children’s Hospital, Nedlands, WA 6009, Australia; shripada.rao@health.wa.gov.au; 3 School of Medicine, University of Western Australia, Crawley, WA 6009, Australia

**Keywords:** probiotics, dose, preterm infant

## Abstract

Probiotics are known to decrease incidences of necrotising enterocolitis, feeding intolerance, late-onset sepsis, and mortality in preterm infants. Administering an adequate dose is important for optimizing the benefits and safety of probiotics. We conducted a systematic review to assess the effect of probiotic dose escalation on clinical outcomes and gut microbiota in preterm neonates. We searched PubMed, EMBASE, EMCARE, Medline, Cochrane Library, Google Scholar, and MedNar databases in July 2023. Three studies were included. In one of the randomized studies (*n* = 149, gestation 27 to 33 weeks), no significant differences in faecal *Lactobacillus* and *Bifidobacterium* counts and clinical outcomes were seen between the high- and low-dose groups. There was a trend towards increased *Lactobacillus* and *Bifidobacterium* counts in the high-dose group. In the other randomized study (*n* = 120, birth weight 500 to 2000 gm), smaller infants (500 to 1000 gm) required higher doses to display *Lactobacillus* in their faeces. The cohort study (*n* = 12, gestation < 33 weeks) showed a trend towards an increase in faecal abundance of bifidobacteria and bacterial diversity in the *B. infantis* group with increasing dose/time. Limited evidence suggests a higher dose might improve gut colonization in preterm infants. Further studies are urgently needed to address this gap in the knowledge considering the increasing use of probiotics for preterm infants.

## 1. Introduction

Preterm infants are at an increased risk of morbidities such as necrotizing enterocolitis (NEC) [1], late-onset sepsis (LOS) [2], and feeding intolerance, which are known to be associated with poor neurodevelopmental outcomes and long-term growth [3,4,5,6,7,8,9].

Evidence from randomized controlled trials (RCTs) (*n* = 60) and non-RCTs (*n* = 30) shows that probiotics significantly reduce the risk of all-cause mortality, NEC ≥ Stage II, LOS, and feeding intolerance in preterm infants [10,11,12]. However, inadequate evidence on important aspects of supplementation, including optimal dose and strains, continues to be a barrier against adopting probiotics in routine practice for preterm infants [13].

Probiotics are defined as “live microorganisms which, when administered in an adequate amount, confer benefits to the host” [14]. This definition does not clarify what an adequate dose is or should be. Pragmatically, it has been defined as the dose shown to be beneficial in a large RCT [15]. A daily dose of 3 × 10^9^ colony forming units (cfu) based on the median dose used in RCTs has been recommended for preterm infants of a very low birth weight (VLBW) born <32 weeks [15].

Administering an adequate dose is important for the optimal response to probiotics. Critical assessment of the current evidence from probiotic dose–response studies in preterm neonates is hence important. The need for such an assessment is supported by the increasing adoption of probiotics for preterm infants and the call for well-designed, strain-specific probiotic dose–response studies in this cohort [16,17].

Given the significance of the underlying issues, we aimed to conduct a systematic review to evaluate the current evidence from such studies in preterm infants to guide clinical practice and research in this field.

## 2. Materials and Methods

Cochrane guidelines were used to conduct this systematic review and it was reported according to the Preferred Reporting Items for Systematic Reviews and Meta-Analysis (PRISMA) statement [18]. It was registered on the “Open Science Forum” (https://doi.org/10.17605/OSF.IO/M4TYH).

### 2.1. Data Sources and Searches

The databases PubMed, EMBASE (through OVID), EMCARE (through OVID), MEDLINE (through OVID), Cochrane Library, and Google Scholar were searched (since inception until June 2023) independently by reviewers CR and GJ under the guidance of senior author SP. Both primary reviewers for the study identification were moderately experienced in systematic reviews and literature search. The strategy of having two experienced reviewers for independent literature search was used to increase the precision of study selection [19]. The ClinicalTrials.gov website was searched to identify ongoing studies. Grey literature was searched on the Mednar (http://mednar.com/mednar/desktop/en/search.html (accessed on 5 July 2023)) database. Abstracts from the meetings of the Paediatric Academic Society (PAS), and conference proceedings including those from the Perinatal Society of Australia and New Zealand (PSANZ) and the European Academy of Paediatric Societies, were searched and reviewed from 2017. Pubmed was searched using the following keywords: ((“probiotic s” [All Fields] OR “probiotical” [All Fields] OR “probiotics” [MeSH Terms] OR “probiotics” [All Fields] OR “probiotic” [All Fields]) AND “dose” [All Fields] AND (“infant, newborn” [MeSH Terms] OR (“infant” [All Fields] AND “newborn” [All Fields]) OR “newborn infant” [All Fields] OR “neonatal” [All Fields] OR “neonate” [All Fields] OR “neonates” [All Fields] OR “neonatality” [All Fields] OR “neonatals” [All Fields] OR “neonates” [All Fields])) AND (clinical trial [Filter] OR randomized controlled trial [Filter]). Similar terms were used to search other databases. We hand searched the reference lists of relevant articles without applying time or language restrictions. The references identified from the database search were exported to EndNote software (Version 20.6). Duplicate articles were removed, and the full texts of the eligible studies were obtained after reading the abstracts. The full-text articles were read independently by reviewers CR and GJ to assess their suitability for inclusion. Discrepancies in the study selection were resolved by discussions with the senior author.

### 2.2. Study Selection

We included RCTs, quasi-RCTs, and non-RCTs that evaluated the response to sequentially increasing doses of single- or multi-strain probiotics in preterm infants born before 37 weeks of gestation. Studies assessing interventions that included probiotics with other interventions (e.g., prebiotics) were included only if all participants received an equal dose of the cointervention. We included studies where infants were fed their own mother’s milk, donor milk, or formula. Reviews, case reports, letters, editorials, and commentaries were excluded but read to identify potential studies for inclusion.

The primary outcomes included faecal microbiota composition (richness, abundance, evenness, α-diversity or compositional dissimilarity (β-diversity), and short-chain fatty acid (SCFA) levels. The secondary outcomes included NEC ≥ Stage II LOS, defined as a positive blood or cerebrospinal fluid culture after 72 h of life, all-cause mortality, feeding intolerance, duration of parenteral nutrition, time to full feeds, long-term neurodevelopmental outcomes based on validated scales assessed at ≥12 months of corrected age, adverse effects including probiotic sepsis, and any other outcomes reported in the included studies.

### 2.3. Data Extraction and Quality Assessment

A pre-prepared standardised form was used to extract data. Reviewers CR and GJ independently completed the data collection forms. The data on primary and secondary outcomes were abstracted from the included studies. We recorded the adjusted or unadjusted odds ratios (ORs) or risk ratios (RRs) for the outcomes if available in the included studies. For non-RCTs, the modified Newcastle–Ottawa Scale (NOS) [20] was used to assess the risk of bias (ROB) across 8 domains. The quality of the RCTs was assessed using the Cochrane ROB II tool [21].The certainty of evidence was assessed using the GRADE methodology and classified into one of the four categories: high, moderate, low, and very low [22]. In case of discrepancies, group discussions were held involving all authors for reaching consensus.

### 2.4. Data Synthesis

We planned to conduct meta-analysis using Review Manager v5.4 (Cochrane Collaboration, Nordic Cochrane Centre, Copenhagen, Denmark) using a random effects model anticipating heterogeneity. We planned to pool the adjusted and unadjusted ORs separately. Effect size was to be expressed as RR and 95% confidence interval (CI) for dichotomous outcomes, and mean difference and 95% CI for continuous outcomes. Qualitative synthesis was planned if meta-analysis was not possible. We planned to assess heterogeneity using the I^2^ statistic. The I^2^ results were interpreted as follows: 0–40%: might not be important; 30–60%: may represent moderate heterogeneity; 50–90%: may represent substantial heterogeneity; 75–100%: considerable heterogeneity (Cochrane Handbook) [23]. The pre-stated subgroups included analyses based on study design (e.g., RCTs vs. non-RCTs), and gestation <28 weeks. We planned a sensitivity analysis after excluding studies with a high ROB.

## 3. Results

**Figure 1:**
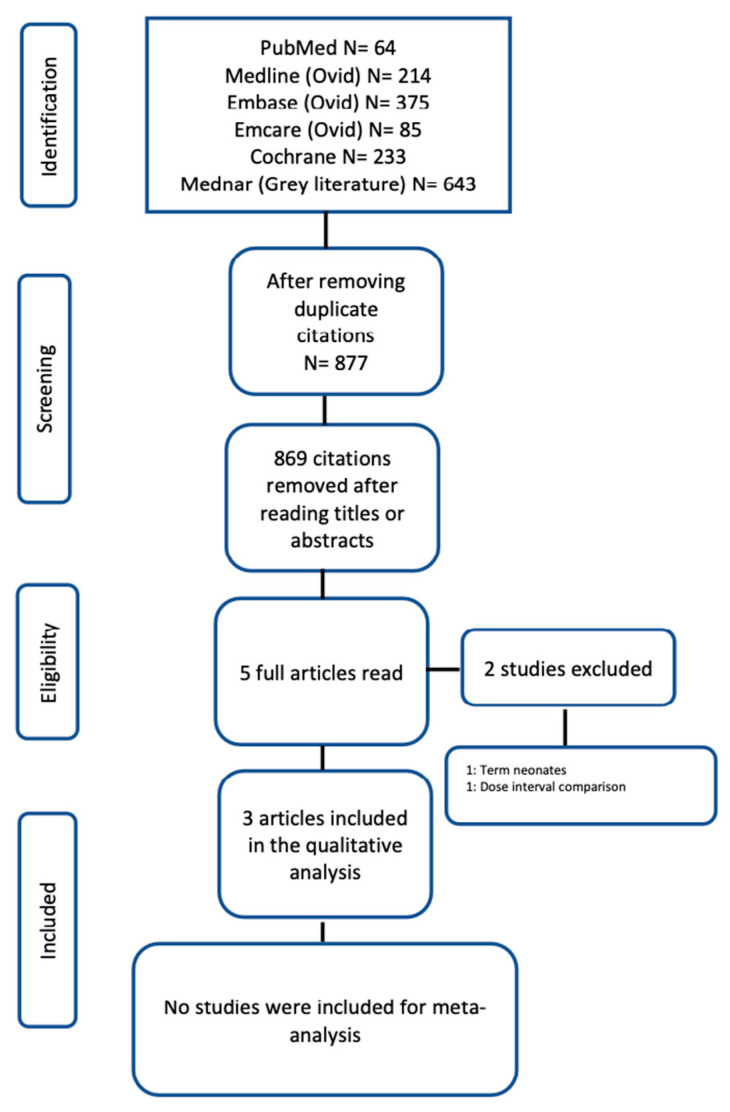
PRISMA flow chart for study selection.

The PRISMA flow chart showing methods of selecting and screening studies is shown in Figure 1. The preliminary search yielded 1614 citations, out of which 3 studies were included in the systematic review [24,25,26]. One of the included studies was a poster sighted in an online portal [26]. The total sample size of these two studies was 281. Two of the included studies were RCTs [24,26] and the third was a cohort study [25]. Two studies were excluded: one that involved the term neonates [27] and another that primarily assessed probiotic dose intervals [28].

One of the included RCTs enrolled 149 preterm infants born between 27 and 33 weeks of gestation. These infants were randomly allocated to different groups based on the following protocol for probiotic or placebo administration: (A): High dose and long course (10^10^ cells 12 hourly for 21 days), (B): High dose and short course (10^10^ cells 12 hourly for days 1–14; followed by placebo from days 15–21), (C): Low dose and long course (10^9^ cells 12-hourly for 21 days), or group (D): Placebo for 21 days. The probiotic sachets contained a mixture of *Lactobacillus* (L.) *acidophilus*, *L. rhamnosus* (LGG), *Bifidobacterium* (B.) *longum*, and *Saccharomyces boulardii*. Stool samples were collected on days 14, 21, and 28 after randomization. The rates of faecal colonization with *Lactobacillus* and *Bifidobacterium* species were significantly different on day 14 and day 28 between the probiotic and placebo groups, but similar between groups A, B, and C (*p* = 0.53). Quantitative colonization rates were not significantly different between groups A, B, and C. However, there was a trend towards a greater number of cfu per ml of stool with *Lactobacillus* and *Bifidobacterium* species in group A vs. B, and group B vs. C. There were no significant differences in clinical outcomes including mortality, NEC ≥ stage II, sepsis, and feeding intolerance between the groups (NEC ≥ stage II: A 1/38, B 3/38, C 2/38 and D 0/35, *p* = 0.49 for group A vs. B vs. C, mortality: A 3/38, B 3/38, C 2/38, D 2/35, *p* = 0.63 for group A vs. B vs. C, culture-positive sepsis: A 3/38, B 1/38, C 6/38, D 6/35, *p* = 0.13 for group A vs. B vs. C). Multivariate logistic regression analysis showed that allocation to the high-dose groups (A or B) was an independent predictor of high (greater than median) *Lactobacillus* counts on day 28 (aOR (95% CI) [Group A *Lactobacillus*: 7.5 (2.1 to 26.5), *p* = 0.002 and group B: 4.9 (1.5 to 16), *p* = 0.008)]. There were no cases of probiotic sepsis reported.

The other included RCT involved 120 preterm neonates (gestation ≤ 32 weeks + 6 days) randomized to receive either a high (10^9^ cfu/day) or low dose (10^8^ cfu/day) of a probiotic (IBP-9414: *Lactobacillus reuteri*) or placebo [26]. Both the dose regimes were well tolerated by the infants, with no significant adverse effects for up to 6 months after the last dose. Quantitative PCR assessment showed that the smaller infants (500 to 1000 gms) required higher doses to display a significant difference in faecal IBP-9414 compared to the placebo. There was no case of sepsis due to the administered probiotic being defined as the isolation of IBP-9414 bacteria from a normally sterile body fluid such as the blood or cerebrospinal fluid.

The small cohort study by Underwood et al. [25] involved 12 preterm formula-fed infants (birth weight < 1500 g, gestation < 33 weeks) randomly allocated to receive increasing doses of either *B. infantis* or *B. lactis* for 5 weeks (week 1: 5 × 10^7^, week 2: 1.5 × 10^8^, week 3: 4.5 × 10^8^, week 4: 1.4 × 10^9^, and week 5: 4.2 × 10^9^ cfu per dose twice a day). Stool specimens were collected at the baseline and then weekly for 5 weeks. Terminal restriction fragment-length polymorphism data did not show large scale shifts in either group. Relative abundance of *Bifidobacteria* decreased with increasing dosage over time in the *B. lactis* group (aOR: 0.69, 95% CI 0.47–0.996). There was a trend towards increase in the *B. infantis* group (aOR: 1.15, 95% CI 0.79–1.69). The change in relative abundance of bifidobacteria over time was close to being statistically significant in the *B. infantis* vs. *B. lactis* group (relative aOR: 1.68, 95% CI 0.99–2.85, *p* = 0.054). The two groups did not differ in the weekly change in the relative abundances of *gamma-proteobacteria* (aOR: 1.12 for weekly change, 95% CI 0.71–1.77). The Shannon diversity scores did not change over time in the *B. lactis* group (weekly increase in diversity score 1.03-fold, 95% CI 0.995–1.06, *p* = 0.09). However, increased diversity over time/dose was seen in the *B. infantis* group (weekly increase 1.12-fold (95% CI 1.01–1.25, *p* = 0.04). There were no cases of probiotic sepsis reported in this study.

**Figure 2:**
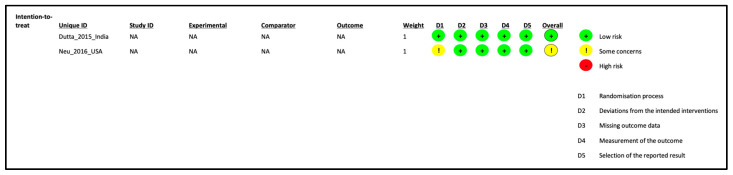
Risk of bias assessment of randomized controlled trials.

Details of the ROB assessment results are provided in Figure 2 and Table 1. The included studies generally had low ROB. The poster on one of the RCTs did not provide randomization details [26]. Meta-analysis was not possible due to the lack of suitable data for pooling. The level of evidence was deemed low, mainly because of the small sample size.

## 4. Discussion

To our knowledge, this is perhaps the first systematic review that evaluated the effect of sequentially increasing doses of probiotics on the faecal microbiota compositions, SCFA levels, and clinical outcomes of preterm neonates. Overall, the findings from the narrative synthesis of the two included studies showed that there was no significant effect of probiotic dose escalation on faecal microbiota and SCFA levels and clinical outcomes in preterm neonates. The meaningfulness of these results is questionable considering they are based on limited data from the included two RCTs (*n* = 269) and one non-RCT (*n* = 12). Furthermore, there was significant heterogeneity in participant characteristics (e.g., gestation, birth weight, type of feeding) and probiotic protocol (strain type, single strain or a mixture, and the range of dosage). The significance of these limitations cannot be overemphasised given that probiotic effects are widely considered to be strain-specific, and the type of feeding (breast milk vs. formula) is an important modifier of probiotic effects [29,30].

The study by Watkins et al. needs to be discussed in the context of probiotic dose–response in preterm neonates [28]. They administered a combination of *B. bifidum* (10^9^ cfu) and *L. acidophilus* (10^9^ cfu) either daily, biweekly, or weekly until 34 weeks postmenstrual age (PMA) to preterm neonates with gestation < 32 weeks [28]. Stool samples were collected at 31, 34, 41 and 44 weeks to look for microbiota composition. *Bifidobacterium* were significantly higher at 31, 34, and 41 weeks of PMA in the daily group compared with the biweekly and weekly (9%) groups [28]. We excluded this study from our systematic review as it assessed the effect of the probiotic dose interval rather than probiotic dose–response using the conventional method of administering sequentially increasing doses of the probiotic. However, it is important to note that the infants in the daily probiotic administration group would have received a higher cumulative dose of the probiotic in this study.

In another neonatal dose de-escalation study, caesarean-born infants on formula feeding were randomized for either standard dose (10^7^ cfu/g of powder) or low-dose (10^4^ cfu/g of powder) *B. lactis* supplementation. The authors reported no statistically significant difference in early life protection against gastrointestinal infections, immune and gut maturation, microbiota establishment, and growth [27]. The outcomes of the high- and low-dose groups were similar to those in the control infants on breast milk.

Given the inadequate evidence from probiotic dose–response studies in preterm infants, it is important to understand the insights from such studies in paediatric and adult populations as well as in animal models. The possibility of an exaggerated pro-inflammatory response in the gastrointestinal tract and an increased risk of bacterial translocation (e.g., probiotic sepsis) at high doses of probiotics are important considerations in this context. This concern is supported by the relatively high pro-inflammatory state of the preterm gut [31,32].

The authors of a dose escalation study that included healthy infants from 0 to 3 months have reported that a low dose (10^8^ cfu/day) of a powdered form of *LGG* was able to provide adequate colonization compared to the medium (10^9^ cfu/day) and high doses (10^10^ cfu/day) [33]. The results of a subgroup analysis (20 studies, *n* = 4038) from a Cochrane review of paediatric antibiotic-associated diarrhoea (AAD) indicated that a high probiotic dose (≥5 billion cfu/day) was more effective than a low dose (<5 billion cfu/day) with an interaction *p* value of 0.01 [34]. The results were considered robust despite the significant dose- and strain-related heterogeneity. In a trial involving children with rotavirus diarrhoea, high- (6 × 10^8^ cfu/day) but not low-dose *LGG* (2 × 10^8^ cfu/day) resulted in statistically significant reductions in faecal rotavirus concentration, showing a dose-dependent effect [35].

Larsen et al. (2006) have reported a dose–response study of *B. animalis* subsp *lactis* BB-12 and *L. paracasei* subsp *paracasei* CRL-341 in healthy young adults [36]. Their results showed increased faecal recovery of *B. lactis* but not *L. paracasei* with increasing doses, suggesting that probiotic dose–response can be strain- or species-specific [36].

The findings of a systematic review by Goodman et al. have shown that probiotics prevent AAD in adults probably by improving dysbiosis and upregulating the innate and adaptive immune systems through various mechanisms [37]. The subgroup analysis from this review showed a significantly reduced risk of AAD (RR 0.54, 95% CI 0.38 to 0.76), *p* = 0.0004) in the higher dose group [37]. Gionotti et al. reported a trend towards probiotic dose–response relationships in their RCT involving adults with colorectal cancer [38]. The dose-related findings in this trial included reduced gut colonization with *Enterobacteriaceae* and increased expression of CD3, CD4, CD8, and naive and memory lymphocyte subsets [38]. It has been hypothesized that SCFAs play an important role in regulating blood pressure and that SCFAs-producing species are known to be lower in individuals with higher blood pressure [39]. A systematic review of studies in adults show that a daily dose of ≥10^11^ cfu probiotics was associated with significant reductions in systolic and diastolic blood pressure [40]. These findings further support the importance of studying probiotic dose–response in the population of interest.

Studies of animal models provide an insight into the interaction between probiotic dose, inflammatory markers, and clinical outcomes. Zhou et al. investigated the dose-dependent effects of *B. longum* in relieving loperamide hydrochloride-induced constipation in rats using colon-released probiotic capsules. The three-strain *B. longum* showed a better ability to control gastrointestinal peptides at a low dose (2.1 ± 0.1 × 10^4^) compared with high (9 ± 0.1 × 10^8^) and medium (9.8 ± 0.2 × 10^6^) doses [41]. High-dose *Bifidobacterium* also improved intestinal motility by improving dysbiosis, SCFA levels, and upregulating the expression of serotonin receptors. It is important to note that intestinal motility, SCFA levels, and the regulation of serotonin receptors play an important role in the pathogenesis of NEC [42,43].

Wen et al. (2015) have reported that administration of five compared to nine doses of LGG significantly reduced the mean duration of rotavirus diarrhoea in gnotobiotic pigs [44]. The two different probiotic dosage groups showed differentially enhanced rotavirus-specific memory B-cell responses, and interferon gamma in different lymphoid tissues (five-dose group in ileum and nine-dose group in spleen). However, only the five-dose group showed significantly improved virus-specific IgA responses in the ileum when challenged. This finding was noted despite any significant differences in faecal *LGG* shedding. The authors hypothesized that the difference in *LGG* dosing frequencies (alternate day vs. daily) may have regulated intestinal antigen-presenting cells differently, leading to different adaptive immune responses [44].

Wen et al. (2012) evaluated the effects of high and low doses of *L. acidophilus* on T-cell immune responses induced by an oral human rotavirus vaccine in gnotobiotic pigs [45]. They reported significantly enhanced effector T-cell responses and reduced regulatory T-cell responses with low-dose (five doses) probiotics. In contrast, significantly downregulated effector T-cell responses and upregulated regulatory T-cell responses were noted with high-dose probiotics (14 doses) [45]. These findings suggest significant implications of dose–response in terms of the immunomodulatory/stimulatory effects of probiotics.

Sun et al. assessed the potential of *L. salivarius* in improving gut health by reducing inflammation and oxidative stress in a dose-dependent manner during weaning in piglets pre-treated with lipopolysaccharide [46]. The high-dose probiotic group showed significantly elevated levels of anti-inflammatory cytokines, tight junction proteins, and downregulated pro-inflammatory mediators in the serum. The expression of TLR2 and TLR4 in the spleen and mesenteric lymph nodes was significantly lower in the high- but not the low-dose group. These findings provide useful insights on probiotic dose–response as the pathophysiology of this model closely resembles that of NEC in preterm infants. The dose–response study of *B. breve* in a mice psoriasis model by Chen et al. showed significantly reduced IL-17 and TNF-alpha levels and improved gut microbiota following a high (10^9^ or 10^10^ cfu) compared with a low dose (10^6^,10^7^ or 10^8^ cfu) of the probiotic. The authors recommended a dose higher than 10^8.42^ cfu/day to improve symptoms of psoriasis according to the dose–effect curve [47]. It is important to note that findings from animal studies may not translate into clinical studies given the species differences. However, despite their limitations, they do provide valuable information for designing high-quality probiotic dose–response studies in preterm infants.

Dose–response studies in the field of probiotics need to be considered in the context of such studies in drugs. Demonstration of a dose–response is considered as strong evidence for a causal relationship between the exposure and the outcome. However, absence of such a response does not rule out a dose–response relationship. The evidence from studies in animals and adult and paediatric populations that we have presented supports that probiotic dose–response is plausible. The effective and safe dose of a probiotic may depend on various factors including probiotic species/strain, disease pathophysiology, age of the subjects, and possibly dose interval. In addition, dose–response relationships can be influenced by the time between the exposure and the measurement of the outcomes. To our knowledge, probiotics dose–response studies have not been reported in extremely preterm infants. There is a possibility that higher doses may improve outcomes. However, they may be associated with adverse effects such as exaggerated gastrointestinal inflammation and probiotic sepsis. It is important to note that probiotics are currently considered to be safe and doses of up to 20 billion per day have been used without any adverse effects [24,48,49].

The strengths and limitations of our review need to be acknowledged. The strengths of our systematic review include the use of robust methodology and a comprehensive literature search. It is important to note that routine use of prophylactic probiotic supplementation for preterm infants is on the rise following the conditional recommendations of expert bodies such as the European Society of Gastroenterology, Hepatology and Nutrition (ESPGHAN) [50,51]. For example, the use of probiotics in tertiary neonatal intensive care units has increased from 17% in 2018 to over 67% in 2022 in the UK [52], and from 14% in 2015 to over 39% in 2022 in the USA [53,54]. The importance of well-designed probiotic dose–response studies in preterm infants cannot be overemphasized considering that an adequate dose is required for optimizing the benefits and safety of probiotics in this high-risk cohort. Our systematic review highlights this critical gap in knowledge. Our findings will help in guiding further research in this field. The limitations of our review include the limited number of studies with a small sample size, heterogeneity in the participant characteristics, probiotics strains and protocols for administration, and a lack of suitable data for pooling.

## 5. Conclusions

Limited evidence suggests that higher probiotic doses might improve gut colonization by potentially beneficial bacteria. The number of probiotic dose–response studies in preterm infants are limited. Well-designed studies are urgently needed in this field to optimize the benefits and safety of probiotics in preterm infants.

## Figures and Tables

**Table 1:** Risk of bias assessment of cohort studies.

Study ID	Selection				Comparability	Outcome			Total Score
	Representativeness of the exposed cohort	Selection of the non-exposed cohort	Ascertainment of exposure	Demonstration that outcome of interest was not present at start of study	Comparability of cohorts on the basis of the design or analysis	Assessment of outcome	Was follow-up long enough for outcomes to occur?	Adequacy of follow up of cohorts	
Underwood_2013_USA	*	*	*	*	**	*	*	*	9

## Data Availability

Data available on request.
